# Genetic liability to insomnia in relation to cardiovascular diseases: a Mendelian randomisation study

**DOI:** 10.1007/s10654-021-00737-5

**Published:** 2021-03-12

**Authors:** Shuai Yuan, Amy M. Mason, Stephen Burgess, Susanna C. Larsson

**Affiliations:** 1 Unit of Cardiovascular and Nutritional Epidemiology, Institute of Environmental Medicine, Karolinska Institutet, Nobelsväg 13, 17177 Stockholm, Sweden; 2 British Heart Foundation Cardiovascular Epidemiology Unit, Department of Public Health and Primary Care, University of Cambridge, Cambridge, UK; 3 National Institute for Health Research Cambridge Biomedical Research Centre, University of Cambridge and Cambridge University Hospitals, Cambridge, UK; 4 Department of Public Health and Primary Care, University of Cambridge, Cambridge, UK; 5 MRC Biostatistics Unit, University of Cambridge, Cambridge, UK; 6 Unit of Medical Epidemiology, Department of Surgical Sciences, Uppsala University, Uppsala, Sweden

**Keywords:** Insomnia, Cardiovascular disease, Mendelian randomisation analysis

## Abstract

The present study aimed to determine the associations between insomnia and cardiovascular diseases (CVDs) using Mendelian randomisation (MR) analysis. As instrumental variables, we used 208 independent single-nucleotide polymorphisms associated with insomnia at the genome-wide significance threshold in a meta-analysis of genome-wide association studies in the UK Biobank and 23andMe including a total of 397 959 self-reported insomnia cases and 933 057 non-cases. Summary-level data for nine CVDs were obtained from the UK Biobank including 367 586 individuals of European ancestry. After correction for multiple testing, genetic liability to insomnia was associated with higher odds of six CVDs, including peripheral arterial disease (odd ratio (OR) 1.22; 95% confidence interval (CI), 1.21, 1.33), heart failure (OR 1.21; 95% CI, 1.13, 1.30), coronary artery disease (OR 1.19; 95% CI, 1.14, 1.25), ischaemic stroke (OR 1.15; 95% CI, 1.06, 1.25), venous thromboembolism (OR 1.13; 95% CI, 1.07, 1.19) and atrial fibrillation (OR 1.10; 95% CI, 1.05, 1.15). There were suggestive associations for aortic valve stenosis (OR, 1.17; 95% CI, 1.04, 1.32) and haemorrhagic stroke (OR 1.14; 95% CI, 1.00, 1.29) but no association for abdominal aortic aneurysm (OR, 1.14, 95% CI, 0.98, 1.33). The patterns of associations remained with mild attenuation in multivariable MR analyses adjusting for genetically correlated phenotypes and potential mediators, including sleep duration, depression, body mass index, type 2 diabetes and smoking. The present MR study suggests potential causal associations of genetic liability to insomnia with increased risk of a broad range of CVDs.

**Supplementary Information:**

The online version contains supplementary material available at 10.1007/s10654-021-00737-5.

## Introduction

Insomnia is the commonest sleep disorder and the second most prevalent mental disorder, and is featured by difficulty initiating or maintaining sleep, early-morning awakenings, or having a feeling of nonrestorative sleep [1, 2]. Cardio-detrimental effects of insomnia have been recognized by prospective observational studies [3–6], especially on coronary heart disease [6–8], heart failure [6, 8, 9], ischemic stroke [7, 8], and atrial fibrillation [8, 10]. However, available data on the role of insomnia in certain cardiovascular diseases (CVDs), such as venous thromboembolism, peripheral arterial disease, aortic valve stenosis and abdominal aortic aneurysm, is limited. In addition, given that the majority of epidemiological evidence on insomnia and CVDs was embedded in an observational design, which is less likely to fully account for confounding and reverse causation bias, the causality of the associations between insomnia and various CVDs remains unestablished. Randomized controlled trials on this topic are impractical due to ethical consideration and exhausting source demand. Thus, whether insomnia can be treated as a potentially modifiable cardiovascular risk factor, which has been proposed by previous literature [11, 12], needs further assessment.

Employing genetic variants as instrumental variables for an exposure (e.g., insomnia), Mendelian randomisation (MR) is an analytical approach empowering the causal inference on an exposure-outcome association by reducing residual confounding and reverse causality [13, 14]. The association obtained from MR analysis is less likely to be biased by confounding since genetic variants are randomly allocated at conception and therefore, the trait is generally not correlated to other traits. This natural randomisation process resembles the random assignment of participants to experimental and control groups in a randomized controlled trial, which means individuals with genetic variants leading to a higher liability to insomnia will on average be exposed to a higher risk of having insomnia compared with their counterparts with the genetic variants related to lower liability to insomnia. The MR analysis also diminish reverse causality as genetic variants are fixed and cannot be modified by onset or progression of CVDs. Hence, if a genetic variant that alters the liability to insomnia is also related to CVDs, this provides strong evidence that the insomnia is a cause of CVDs. As far as we know, the causal associations between insomnia and a broad range of CVDs have not been established using MR. We, therefore, applied an MR framework to determine the causal associations of insomnia with nine CVDs. Given that insomnia was genetically correlated with other phenotypes and potential mediators, including sleep duration, depression, body mass index, type 2 diabetes, and smoking [1], we aimed to disentangle the direct effect of insomnia on CVDs whilst accounting for these factors by using the multivariable MR method.

## Methods and materials

### Study design

**Fig. 1:**
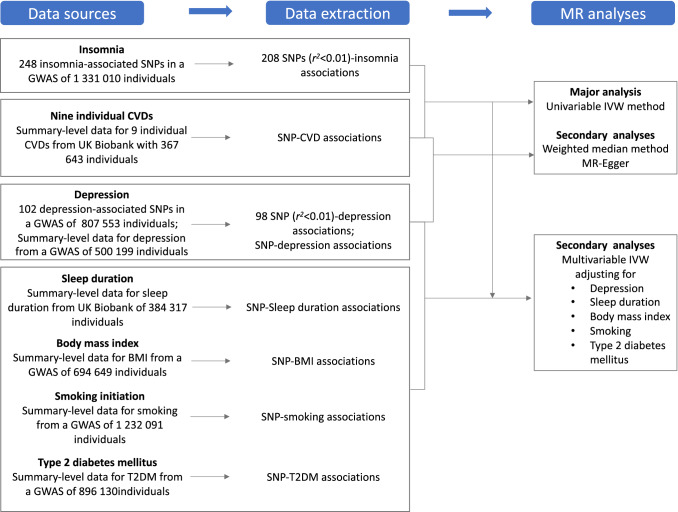
Overview of study design *BMI* indicates body mass index; *CVD*, cardiovascular disease; GWAS genome-wide associations study; *IVW* inverse-variance weighted; *SNP* single-nucleotide polymorphisms. T2DM, type 2 diabetes mellitus. Data extracted were beta coefficients with corresponding standard errors of the SNP–insomnia, SNP-CVDs, SNP-depression, SNP-sleep duration, SNP-BMI, SNP-smoking and SNP-T2DM associations.

Figure 1 shows the overview of study design. Genetic instrument selection was based on a large-scale genome-wide association study (GWAS) for insomnia [1]. Data for the associations of the insomnia-associated SNPs with 9 CVDs were available from UK Biobank [15]. Data for sleep duration [1], depression [16], body mass index [17], type 2 diabetes [18], and smoking initiation [19] were available from corresponding GWASs. Detailed information on data sources used is available in Supplementary Table 1. Several MR methods were used to assess the associations between genetic liability to insomnia and nine CVDs. The UK Biobank study was approved by the North West Multicenter Research Ethics Committee. Original studies included in used GWAS had been approved by a relevant review board. The present analyses were approved by the Swedish Ethical Review Authority.

## Genetic instruments selection

Single-nucleotide polymorphisms (SNPs) associated with insomnia at the genome-wide significance level (*p* < 5 × 10^–8^) were extracted from a meta-analysis of two GWASs including a total of 1,331,010 individuals (386 533 individuals from UK Biobank and 944,477 individuals from 23andMe) of European ancestry [1]. Insomnia was defined by self-reported information, which was collected via a questionnaire in a touchscreen device in UK Biobank and an online questionnaire survey in 23andMe. The questionnaire in the UK Biobank had high sensitivity (98%) and specificity (96%) compared to Insomnia Severity Index or Pittsburgh Sleep Quality Index. The phenotypes in the UK Biobank and 23andMe had acceptable sensitivity and specificity (> 80%) compared with the phenotype defined by structured interview. There were 109,402 cases and 277,131 non-cases in the UK Biobank and 288,557 cases and 655,920 non-cases in 23andMe. Identification of lead SNPs and genomic risk loci was based only on SNPs that were available in both the UK Biobank and 23andMe sample. The GWAS identified 248 lead SNPs, located in 202 loci (> 250 kb apart), explaining ~ 2.6% of the variance in insomnia. The linkage disequilibrium across 248 SNPs were estimated using clump function in the TwoSampleMR package [20] based on the 1000 genomes LD reference panel of only Europeans [21]. Among these, 208 independent SNPs (defined as r^2^ < 0.01 and clump window > 10,000 kb) were proposed as instrumental variables for insomnia. The association tests were adjusted for age, sex, genotype array, and 10 genetic principal components in the UK Biobank, and age, sex and the top five principal components in 23andMe.

## UK biobank

Summary-level statistic for the insomnia-associated SNPs with the 9 CVDs came from the UK Biobank, that is, a cohort study of about 500,000 adults aged 37–73 years and enrolled between 2006 and 2010 [15]. Non-Europeans were excluded to minimize confounding by ancestry. In addition, we excluded individuals with relatedness of third degree or higher, low genotype call rate (three or more standard deviations from the mean) and excess heterozygosity. We had a final study sample of 367 586 individuals of European descent who were followed up until the 31st March 2017 (hospital episode statistics) or the 22nd March 2019 (death certificates). The median follow-up was 8.0 years. CVDs were defined based on electronic health records, hospital procedure codes, and self-reported information validated by interview with a nurse (Supplementary Table 3). The association tests were conducted using logistic regression with adjustment for age, sex, ten genetic principal components to obtain the beta coefficients and standard errors for the SNP-CVD associations.

## Statistical analysis

The inverse-variance weighted model with random-effects was employed as the main statistical analysis. Ratio estimates are calculated for each SNP using the formula: Ratio = beta coefficient for the SNP-CVD association/beta coefficient for the SNP-insomnia association. These estimates are then combined in a random-effects inverse-variance weighted meta-analysis. Estimates from this method have the highest precision and rely on the assumption that all SNPs are valid instrumental variables [22]. We used the *I*^*2*^ statistic to measure the heterogeneity among estimates across individual SNPs [23]. The weighted median and MR-Egger regression approaches were used as sensitivity analyses to test the robustness of the results and correct for pleiotropy. The weighted median method can generate consistent estimates if at least 50% of the weight in the analysis comes from valid instrumental variables [24]. The MR-Egger approach can detect and correct for directional pleiotropy albeit with compromised power [25].

Multivariable MR analyses were conducted to examine the direct effect of insomnia on CVD outcomes whilst accounting for potential mediation or confounding effects by sleep duration, depression, body mass index, type 2 diabetes and smoking, which are genetically correlated with insomnia [1]. Given the strong genetic correlation between insomnia and depression in particular (*r*_*g*_ = 0.5), we conducted an MR analysis to assess the impact of genetic predisposition to depression on nine CVDs in addition to the multivariable MR analysis adjusting for genetic liability to depression. We adopted the multivariable MR model solely for testing mediation by these factors, rather than allowing for independent effects of these factors and their mediations effects simultaneously.

We used the Bonferroni method to correct for multiple testing. The association with two-sided *p*-values < 0.006 (where α = 0.05/9 CVD outcomes) were deemed statistically significant and also considered strong evidence of a causal association. Associations with *p*-values between 0.05 and 0.006 were regarded as suggestive evidence of association. All analyses were performed using the mrrobust package [26] in Stata/SE 15.0 (Stata Statistical Software: Release 15. College Station, TX: StataCorp LLC.) and the TwoSampleMR [20] and MendelianRandomization [27] packages in R Software 3.6.0 (R Core Team. R Foundation for Statistical Computing. Vienna, Austria. 2019. https://www.R-project.org).

## Results

### Basic characteristics

The prevalence of insomnia was 29.9% in the combined population of UK Biobank and 23andMe. Two-thirds of the sample were older than 45 and one-third of participants were older than 60 years of age in 23andMe. The mean age of participants was approximately 57 years and ~ 46% were men in the UK Biobank study. The *F* statistic for the score of 208 instrumental variables was ~ 143.2 in the UK Biobank.

## Association of genetic liability to insomnia with CVDs

**Fig. 2:**
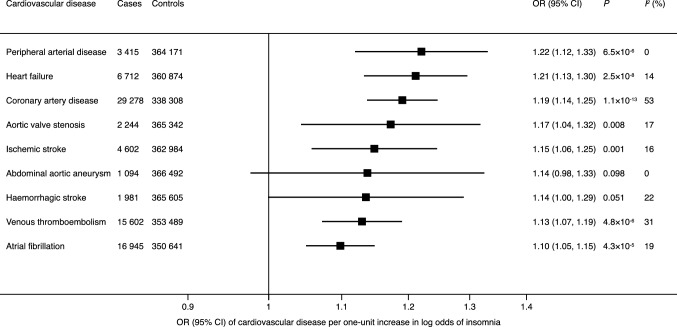
Associations of genetic liability to insomnia with 9 cardiovascular diseases in UK Biobank. *CI* indicates confidence interval; *OR* odds ratio. Estimates are from the random-effects inverse variance-weighted method. The *I*^*2*^ statistic quantifies the amount of heterogeneity among estimates based on individual SNPs. Significant at the Bonferroni-corrected threshold of *p* < 0.006

**Fig. 3:**
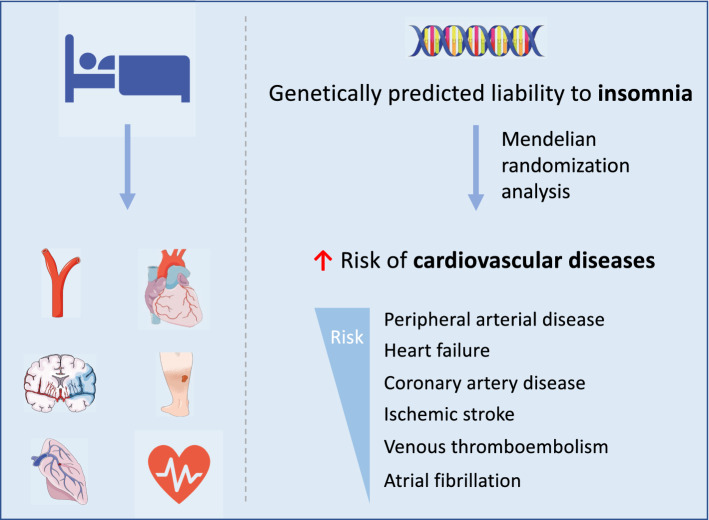
Genetic liability to insomnia in relation to cardiovascular diseases

Genetic liability to insomnia was significantly positively associated with 6 out of 9 outcomes in the main analysis (Figs. 2, 3). For one-unit increase in log odds of insomnia (equalling to one-unit increase in the prevalence of insomnia), the odds ratios were 1.22 (95% CI, 1.21, 1.33) for peripheral arterial disease, 1.21 (95% CI, 1.13, 1.30) for heart failure, 1.19 (95% CI, 1.14, 1.25) for coronary artery disease, 1.15 (95% CI, 1.06, 1.25) for ischaemic stroke, 1.13 (95% CI, 1.07, 1.19) for venous thromboembolism and 1.10 (95% CI, 1.05, 1.15) for atrial fibrillation. There were suggestive associations of genetic liability to insomnia with aortic valve stenosis (OR, 1.17; 95% CI, 1.04, 1.32) and haemorrhagic stroke (OR 1.14; 95% CI, 1.00, 1.29), but limited evidence supporting a causal association of genetic liability to insomnia with abdominal aortic aneurysm (OR, 1.14, 95% CI, 0.98, 1.33). Results remained consistent in the sensitivity analysis using the weighted median method, but possible pleiotropy was detected in the analyses of haemorrhagic stroke and venous thromboembolism (Supplementary Table 4). After correcting for pleiotropy in the MR-Egger analysis, genetic liability to insomnia showed a suggestive positive association with haemorrhagic stroke (OR 2.06; 95% CI, 1.25, 3.39).

Genetic liability to depression was significantly associated with coronary artery disease and heart failure (Supplementary Fig. 1). In the multivariable MR analysis adjusting for depression, the magnitude of associations of genetic liability to insomnia with CVDs attenuated and was only statistically significant at the Bonferroni corrected *p* value for aortic valve stenosis, coronary artery disease and heart failure (Supplementary Fig. 2). Likewise, after adjustment for sleep duration, most associations between genetic liability to insomnia and CVDs attenuated slightly and only the associations with peripheral artery disease, heart failure and coronary artery disease remained significant at the Bonferroni-corrected significance level (Supplementary Fig. 3).

Genetic liability to insomnia was positively associated with body mass index, smoking and type 2 diabetes (Supplementary Fig. 4). In the multivariable MR analysis adjusting for these genetically correlated phenotypes, the effect sizes of associations attenuated mildly, whereas the overall patterns for the associations of insomnia with CVD outcomes remained (Supplementary Fig. 5). The association with aortic valve stenosis did not persist in the multivariable MR analysis adjusting for body mass index.

## Discussion

### Principal findings

The present study based on genetic data for 367 586 women and men found that genetic liability to insomnia was associated with higher risk of a broad range of CVDs. The strongest associations were observed for peripheral arterial disease, heart failure, coronary artery disease, ischaemic stroke, venous thromboembolism and atrial fibrillation. There were suggestive associations for aortic valve stenosis and haemorrhagic stroke but no association for abdominal aortic aneurysm. The pattern of associations remained after adjustment for genetically predicted sleep duration and genetic liability to depression. Genetic predisposition to insomnia appeared to influence body mass index, type 2 diabetes and smoking in an unfavourable manner and these factors might partly mediate the link from insomnia to these CVDs.

## Previous studies

The majority of previous observational studies identified that insomnia was associated with increased risk of overall CVDs in both Western and Eastern populations [3, 5, 6], which was supported by the present MR study. When looking at specific CVD outcomes, our results were also in agreement with the findings suggesting that insomnia is a risk factor for coronary heart disease [3, 6–8], heart failure [6, 8, 9], ischemic stroke [3, 7, 8] and atrial fibrillation [8, 10]. A previous MR study using data from genetic consortia found a major influence of insomnia on four common CVDs, including coronary artery disease, heart failure, atrial fibrillation and ischemic stroke [8]. Our findings were directionally consistent with the previous MR study, but the estimates were of somewhat stronger magnitude. The reason behind the discrepancy in effect size might be a more consistent case definitions in UK Biobank, dilution effects introduced by population mixture in large consortia or differences in insomnia status and prevalence of other risk factors in different populations.

Epidemiological evidence on the associations of insomnia with peripheral arterial disease, venous thromboembolism and abdominal aortic aneurysm is scarce. Mechanistic findings may lend supports to these novel associations. In detail, studies revealed a higher level of von Willebrand factor [28] and inflammation [29] that facilitate the process of coagulation and atherosclerosis among individuals with shorter sleep duration. Although we noticed a general adverse impact of insomnia on CVDs, the present study provided limited evidence in support of causal associations of insomnia with aortic valve stenosis, haemorrhagic stroke and abdominal aortic aneurysm. The possible reason may be small number of cases of these outcomes, and therefore, inadequate power in these analyses.

Insomnia had a high genetic correlation with depression [1], which may pose an overall risk on CVDs based on a mechanistic analysis [30] and may be a confounder in observed association between insomnia and CVDs. Nonetheless, except for effects on coronary artery disease and heart failure, the present and a previous MR study [31] did not reveal any causal associations between genetic liability to depression and CVDs. After adjusting for genetic predisposition to depression, the associations of insomnia with coronary artery disease and heart failure remained and the magnitude of the association for heart failure became even stronger, which suggests genetically correlated depression with insomnia is less likely to be a source biasing the associations between insomnia and CVDs. Similarly, the pattern of associations of genetic liability to insomnia with CVDs remained after adjustment for genetically predicted sleep duration, which might indicate our findings was less likely to be covered by the genetic correlation between insomnia and sleep duration.

Genetic liability to insomnia appeared to exert a causal effect on body mass index, type 2 diabetes and smoking in an unfavourable manner. These factors might partly mediate the associations between insomnia and CVDs. We found some support for this as the associations between genetic liability to insomnia and CVDs were slightly attenuated in multivariable MR analyses with adjustment for these factors.

## Potential mechanisms

Detailed pathophysiological bases behind the association between insomnia and CVDs are not fully understood. There are several hypotheses supporting such link. At the macro level, insomnia was found to be causally associated with several risk factors in a bad manner, such as body mass index, smoking and type 2 diabetes in the present and previous study. Increased body mass index [32], smoking [33] and type 2 diabetes [34] were identified to possibly trigger CVDs. At the micro level, insomnia is a disorder of hyperarousal with chronic activation of stress responses consequently accompanied by increased metabolic rate, increased heart rate and decreased heart rate variability, and increased cortisol secretion. Such insomnia-derived abnormalities in the autonomic nervous system and hypothalamic pituitary axis may partly explain the increased risk of CVDs among insomnia patients [35]. The elevated blood pressures among insomnia patients may also partly solidify the causal association linking insomnia to cardiovascular disease [36]. In addition, insomnia itself can induce alternations in inflammation, oxidative stress, and dyslipidaemia, which further lead to an increase in arterial blood pressure, endothelial dysfunction, diabetes mellitus, and accelerated atherosclerosis [12].

## Validation of assumptions of MR

There are three assumptions of MR that need to be satisfied to determine causality in an MR analysis. First, the genetic variants used as instrumental variables should be robustly associated with the risk factor, in this case, insomnia. In the present study, genetic instruments associated with insomnia were selected from a large-scale GWAS including 1 331 010 individuals at *p* < 5 × 10^–8^. Second, the selected genetic variants should not be associated with potential confounders. Even though genetic instruments for insomnia were associated with sleep duration and depression that might be confounders, the multivariable MR analysis adjusting for these factors revealed that the pattern of associations of insomnia with CVD persisted. Thirdly, the genetic variants should affect the risk of the outcome, in this case CVD, only through the risk factor, not via alternative pathways. This assumption was also satisfied by conducting multivariable MR analyses adjusting for depression, smoking and type 2 diabetes. These analyses suggested that depression, smoking and type 2 diabetes may be mediators of the relationship between insomnia and CVD.

## Strengths and limitations

Our study has several strengths. The major one is the MR design, which reduced the possibility that the observed associations were biased by confounding and other biases. The associations of insomnia with type 2 diabetes and coronary artery disease were revealed in the GWAS on insomnia [1] and successfully replicated in the present study indicating the genetic instrument for insomnia robustly associated with insomnia, thereby providing a strong genetic instrument. In addition, the statistical power was high in analyses of prevalent cardiovascular outcomes in UK Biobank. Finally, we only included participants of European descent in the exposure and outcome datasets, so population stratification bias was minimized. However, this population confinement might on the other hand limit the generalizability of our findings to other populations.

The present study also has several limitations. First, we could not completely expel the possibility that the insomnia-related SNPs affect CVDs through other causal pathways than through insomnia. However, major genetically correlated traits, such as sleep duration and depression were adjusted for in multivariable MR analyses and the associations remained stable with slight attenuation after the adjustment. Second, we might have missed weak associations given the small number of cases for certain CVDs, in particular haemorrhagic stroke and abdominal aortic aneurysm. Another limitation is that there was a partial (approximately 27.6%) overlap between the exposure (insomnia) and outcome (CVD) datasets, which could lead to model overfitting [37]. This limitation might bias the causal estimates towards the direction of the observational association between insomnia and CVD risk [37]. However, an *F-*statistic > 10 for the genetic risk score revealed that the used instrumental variables are strongly associated with the exposure [37]. In addition, our results were in agreement with findings based on large consortia data with less or no overlap [8]. Thus, any potential bias due to sample overlap is expected to be small.

## Conclusions

The present MR study reveals evidence supporting causal associations of genetic liability to insomnia with a broad range of CVDs, in particular peripheral arterial disease, heart failure, coronary artery disease, ischaemic stroke, venous thromboembolism and atrial fibrillation. Strategies to reduce insomnia may be one of the cornerstones in the prevention of CVDs.

## Supplementary Information

Below is the link to the electronic supplementary material.

Supplementary file1 (DOCX 3149 KB)

## Data Availability

Data can be obtained by a reasonable request to corresponding author.
