# Supplementary material for: Genetic liability to insomnia in relation to cardiovascular diseases: a Mendelian randomisation study
Source: Eur J Epidemiol. 2021 Mar 12;36(4):393–400. doi: 10.1007/s10654-021-00737-5 (PMC8076146; doi:10.1007/s10654-021-00737-5)
Supplement: Supplementary file 1 — Supplementary file1 (DOCX 3149 KB) [file 10654_2021_737_MOESM1_ESM.docx]

**Supplements**

**Genetic liability to insomnia in relation to cardiovascular disease: a Mendelian randomisation study**

*Shuai Yuan, Amy M. Mason, Stephen Burgess, Susanna C. Larsson*

**Supplementary Table 1**. Detailed information on included traits in the present study

**Supplementary Table 2.** Detailed information on genetic instruments

**Supplementary Table 3**. Definition and sources of information for cardiovascular disease outcomes in UK Biobank

**Supplementary Table 4**. Associations of genetically predicted liability to insomnia with 9 individual cardiovascular diseases in UK Biobank in sensitivity analyses

**Supplementary Figure 1.** Associations of genetically predicted liability to depression with 9 individual cardiovascular diseases in UK Biobank

**Supplementary Figure 2.** Associations of genetically predicted liability to insomnia with 9 individual cardiovascular diseases after adjusting for depression in UK Biobank

**Supplementary Figure 3.** Associations of genetically predicted liability to insomnia with 9 individual cardiovascular diseases after adjusting for sleep duration in UK Biobank

**Supplementary Figure 4.** Associations of genetically predicted liability to insomnia with body mass index, smoking, alcohol drinking and type 2 diabetes mellitus

**Supplementary Figure 5.** Associations of genetically predicted liability to insomnia with 9 individual cardiovascular diseases after adjusting for body mass index, smoking and type 2 diabetes mellitus in UK Biobank

**Supplementary Table 1**. Detailed information on included traits in the present study

| **Traits** | **Data source** | **Sample size** | **Web link for data source** |
| --- | --- | --- | --- |
| Insomnia | UK Biobank and 23andMe | 1 331 010 individuals (386 533 from UK Biobank and 944 477 from 23andMe) | <https://ctg.cncr.nl/software/summary_statistics> |
| Cardiovascular disease | UK Biobank | 367 643 individuals | <https://www.ukbiobank.ac.uk/> |
| Depression (SNPs) | UK Biobank, 23andMe and Psychiatric Genomics Consortium | 807 553 individuals (246 363 cases and 561190 non-cases) | <https://datashare.is.ed.ac.uk/handle/10283/3203> |
| Depression (summary-level) | UK Biobank and Psychiatric Genomics Consortium | 500 199 (170 756 cases and 329 443 non-cases) | <https://datashare.is.ed.ac.uk/handle/10283/3203> |
| Sleep duration | UK Biobank | 384 317 individuals | <https://ctg.cncr.nl/software/summary_statistics> |
| Body mass index | GIANT consortium | 694 649 individuals | <https://zenodo.org/record/1251813#.Xbhf9pNKiqB> |
| Smoking initiation | 29 studies | 1 232 091 individuals | <https://conservancy.umn.edu/handle/11299/201564> |
| Alcohol drinking | 29 studies | 941 280 individuals | <https://conservancy.umn.edu/handle/11299/201564> |
| Type 2 diabetes mellitus | DIAGRAM consortium | 898 130 individuals (74 124 cases and 824 006 non-cases) | [https://www.diagram-consortium.org](https://www.diagram-consortium.org/) |

DIAGRAM indicates DIAbetes Genetics Replication And Meta-analysis; GIANT, Genetic Investigation of ANthropometric Traits.

**Supplementary Table 2.** Detailed information on genetic instruments

| **rsID** | **Chr** | **Position** | **EA** | **NEA** | **EAF** | **Beta** | **SE** | ***P-value*** |
| --- | --- | --- | --- | --- | --- | --- | --- | --- |
| rs2089358 | 1 | 37194103 | C | T | 0.30 | 0.041 | 0.007 | 2.75E-10 |
| rs11588755 | 1 | 57819204 | G | A | 0.48 | 0.035 | 0.006 | 5.14E-09 |
| rs1937447 | 1 | 66358242 | G | C | 0.24 | 0.039 | 0.007 | 2.08E-08 |
| rs1620977 | 1 | 72729142 | A | G | 0.27 | 0.052 | 0.007 | 2.27E-14 |
| rs699844 | 1 | 74878253 | A | G | 0.92 | 0.060 | 0.011 | 4.11E-08 |
| rs12030482 | 1 | 96961268 | A | T | 0.22 | 0.041 | 0.007 | 8.16E-09 |
| rs6702604 | 1 | 107190062 | G | A | 0.42 | 0.037 | 0.006 | 1.30E-09 |
| rs1289939 | 1 | 117944435 | C | T | 0.77 | 0.041 | 0.007 | 6.00E-09 |
| rs5877 | 1 | 173878862 | T | C | 0.67 | 0.036 | 0.006 | 1.23E-08 |
| rs11803128 | 1 | 190060095 | G | A | 0.35 | 0.041 | 0.006 | 6.85E-11 |
| rs10800992 | 1 | 190900576 | T | C | 0.44 | 0.042 | 0.006 | 3.84E-12 |
| rs623025 | 1 | 201765094 | C | T | 0.74 | 0.038 | 0.007 | 3.16E-08 |
| rs11119409 | 1 | 210293333 | C | T | 0.41 | 0.035 | 0.006 | 1.19E-08 |
| rs823247 | 2 | 2850540 | C | T | 0.52 | 0.037 | 0.006 | 5.25E-10 |
| rs6734957 | 2 | 42813247 | G | T | 0.76 | 0.042 | 0.007 | 1.82E-09 |
| rs56097173 | 2 | 44262449 | T | C | 0.68 | 0.040 | 0.006 | 2.69E-10 |
| rs13010288 | 2 | 51824512 | G | T | 0.87 | 0.060 | 0.009 | 9.26E-12 |
| rs1861412 | 2 | 58893065 | A | G | 0.43 | 0.038 | 0.006 | 1.67E-10 |
| rs6545798 | 2 | 60521311 | T | A | 0.59 | 0.041 | 0.006 | 1.19E-11 |
| rs1519102 | 2 | 66677816 | G | C | 0.31 | 0.037 | 0.006 | 1.90E-08 |
| rs113851554 | 2 | 66750564 | T | G | 0.05 | 0.206 | 0.014 | 1.56E-51 |
| rs75452188 | 2 | 67134426 | A | G | 0.88 | 0.052 | 0.009 | 1.58E-08 |
| rs12991815 | 2 | 68071990 | C | G | 0.42 | 0.040 | 0.006 | 3.02E-11 |
| rs11679943 | 2 | 77724624 | A | G | 0.35 | 0.037 | 0.006 | 3.16E-09 |
| rs72820274 | 2 | 104412924 | A | G | 0.42 | 0.034 | 0.006 | 1.28E-08 |
| rs62158170 | 2 | 114082175 | A | G | 0.79 | 0.066 | 0.007 | 1.20E-19 |
| rs10928256 | 2 | 146458738 | T | C | 0.42 | 0.034 | 0.006 | 1.61E-08 |
| rs6756610 | 2 | 147480394 | C | G | 0.63 | 0.037 | 0.006 | 1.14E-09 |
| rs116466468 | 2 | 159137557 | T | C | 0.76 | 0.044 | 0.007 | 2.11E-10 |
| rs4664299 | 2 | 160570033 | C | T | 0.77 | 0.041 | 0.007 | 4.95E-09 |
| rs7571486 | 2 | 176473295 | G | A | 0.75 | 0.039 | 0.007 | 1.40E-08 |
| rs55772859 | 2 | 208042581 | A | C | 0.31 | 0.042 | 0.006 | 4.82E-11 |
| rs62213452 | 2 | 210380152 | T | G | 0.28 | 0.037 | 0.007 | 2.39E-08 |
| rs34967082 | 2 | 215382654 | A | G | 0.41 | 0.035 | 0.006 | 4.34E-09 |
| rs1530938 | 2 | 236900633 | A | G | 0.44 | 0.036 | 0.006 | 8.82E-10 |
| rs7599697 | 2 | 239231477 | C | T | 0.64 | 0.037 | 0.006 | 5.00E-09 |
| rs6808140 | 3 | 10581380 | T | C | 0.51 | 0.039 | 0.006 | 5.35E-11 |
| rs7615602 | 3 | 18718055 | G | C | 0.73 | 0.040 | 0.007 | 2.59E-09 |
| rs4858708 | 3 | 25154112 | T | A | 0.47 | 0.034 | 0.006 | 1.23E-08 |
| rs35110063 | 3 | 43066558 | A | G | 0.43 | 0.039 | 0.006 | 8.82E-11 |
| rs7625896 | 3 | 44062561 | A | G | 0.65 | 0.036 | 0.006 | 5.28E-09 |
| rs10865954 | 3 | 49211989 | T | C | 0.33 | 0.042 | 0.006 | 1.92E-11 |
| rs3774751 | 3 | 50209053 | G | T | 0.54 | 0.041 | 0.006 | 7.32E-12 |
| rs1567084 | 3 | 71435955 | A | G | 0.50 | 0.033 | 0.006 | 2.14E-08 |
| rs17025198 | 3 | 88001713 | A | G | 0.20 | 0.041 | 0.007 | 2.19E-08 |
| rs1580173 | 3 | 107955515 | A | G | 0.56 | 0.033 | 0.006 | 2.28E-08 |
| rs62264767 | 3 | 117642005 | A | C | 0.85 | 0.065 | 0.008 | 1.63E-14 |
| rs492858 | 3 | 155432229 | C | T | 0.92 | 0.066 | 0.011 | 3.46E-09 |
| rs2364921 | 3 | 158522463 | C | T | 0.53 | 0.034 | 0.006 | 2.13E-08 |
| rs694786 | 3 | 173112907 | C | T | 0.54 | 0.044 | 0.006 | 1.97E-13 |
| rs4260410 | 3 | 178469932 | T | C | 0.33 | 0.034 | 0.006 | 4.87E-08 |
| rs2216427 | 3 | 180785697 | C | G | 0.65 | 0.035 | 0.006 | 1.60E-08 |
| rs62301574 | 4 | 22050165 | G | C | 0.20 | 0.042 | 0.007 | 1.37E-08 |
| rs16990210 | 4 | 34720226 | C | T | 0.15 | 0.046 | 0.008 | 1.97E-08 |
| rs17005118 | 4 | 82288564 | A | G | 0.26 | 0.042 | 0.007 | 6.13E-10 |
| rs72657797 | 4 | 90820809 | C | T | 0.82 | 0.056 | 0.008 | 1.52E-12 |
| rs13135092 | 4 | 103198082 | G | A | 0.08 | 0.089 | 0.011 | 2.53E-16 |
| rs4699157 | 4 | 106055212 | C | T | 0.04 | 0.081 | 0.015 | 3.98E-08 |
| rs11722569 | 4 | 112822731 | T | C | 0.66 | 0.034 | 0.006 | 2.91E-08 |
| rs13138995 | 4 | 148987430 | A | G | 0.39 | 0.034 | 0.006 | 1.97E-08 |
| rs17223714 | 5 | 50492629 | A | G | 0.79 | 0.046 | 0.007 | 2.44E-10 |
| rs12520974 | 5 | 61514611 | C | T | 0.52 | 0.036 | 0.006 | 1.69E-09 |
| rs701394 | 5 | 80296487 | G | A | 0.36 | 0.036 | 0.006 | 6.83E-09 |
| rs16903122 | 5 | 87693561 | T | C | 0.25 | 0.055 | 0.007 | 9.04E-16 |
| rs35539975 | 5 | 91607148 | A | G | 0.78 | 0.042 | 0.007 | 4.49E-09 |
| rs17083297 | 5 | 92995477 | C | A | 0.82 | 0.044 | 0.008 | 1.60E-08 |
| rs12187443 | 5 | 102660400 | T | C | 0.67 | 0.040 | 0.006 | 1.64E-10 |
| rs2431108 | 5 | 103947968 | C | T | 0.33 | 0.053 | 0.006 | 7.83E-17 |
| rs152555 | 5 | 106849674 | G | A | 0.15 | 0.052 | 0.008 | 4.83E-10 |
| rs17367725 | 5 | 107112116 | C | T | 0.65 | 0.036 | 0.006 | 9.29E-09 |
| rs8180457 | 5 | 107209814 | C | T | 0.84 | 0.056 | 0.008 | 1.12E-11 |
| rs55972276 | 5 | 135653737 | A | C | 0.14 | 0.073 | 0.009 | 4.19E-17 |
| rs6888135 | 5 | 141254063 | A | C | 0.50 | 0.038 | 0.006 | 1.21E-10 |
| rs4502882 | 5 | 153093998 | C | T | 0.34 | 0.039 | 0.006 | 7.96E-10 |
| rs62383308 | 5 | 165460085 | G | A | 0.92 | 0.060 | 0.011 | 3.98E-08 |
| rs6601080 | 5 | 179511043 | A | G | 0.68 | 0.035 | 0.006 | 2.21E-08 |
| rs11756035 | 6 | 18843810 | C | G | 0.13 | 0.051 | 0.009 | 1.29E-08 |
| rs138678612 | 6 | 30932223 | G | A | 0.02 | 0.117 | 0.020 | 1.41E-08 |
| rs3131638 | 6 | 31475127 | G | A | 0.77 | 0.044 | 0.007 | 7.88E-10 |
| rs10947428 | 6 | 33647058 | C | T | 0.21 | 0.068 | 0.007 | 9.06E-21 |
| rs6457796 | 6 | 34828553 | C | T | 0.27 | 0.039 | 0.007 | 1.12E-08 |
| rs10947690 | 6 | 37631768 | G | A | 0.26 | 0.047 | 0.007 | 4.04E-12 |
| rs9394502 | 6 | 38452503 | C | T | 0.67 | 0.054 | 0.006 | 7.76E-18 |
| rs10947987 | 6 | 41754370 | C | T | 0.56 | 0.033 | 0.006 | 4.08E-08 |
| rs10944696 | 6 | 94498850 | G | A | 0.70 | 0.038 | 0.007 | 7.99E-09 |
| rs2388840 | 6 | 99598756 | G | A | 0.42 | 0.037 | 0.006 | 1.37E-09 |
| rs9373590 | 6 | 101212001 | A | T | 0.51 | 0.040 | 0.006 | 2.18E-11 |
| rs314281 | 6 | 105400605 | C | T | 0.55 | 0.043 | 0.006 | 6.03E-13 |
| rs728017 | 6 | 124292594 | G | A | 0.61 | 0.035 | 0.006 | 9.51E-09 |
| rs62429521 | 6 | 140324582 | A | C | 0.15 | 0.051 | 0.008 | 1.78E-09 |
| rs1147852 | 6 | 147980909 | A | G | 0.31 | 0.039 | 0.006 | 9.94E-10 |
| rs4709655 | 6 | 163280204 | C | T | 0.88 | 0.054 | 0.009 | 3.09E-09 |
| rs117152417 | 6 | 166411281 | G | A | 0.99 | 0.147 | 0.026 | 2.82E-08 |
| rs6978112 | 7 | 1966841 | T | C | 0.41 | 0.034 | 0.006 | 2.11E-08 |
| rs940780 | 7 | 3323848 | T | C | 0.36 | 0.038 | 0.006 | 8.50E-10 |
| rs190073 | 7 | 10985188 | G | A | 0.59 | 0.034 | 0.006 | 2.86E-08 |
| rs2030672 | 7 | 21687925 | C | G | 0.56 | 0.034 | 0.006 | 1.10E-08 |
| rs521484 | 7 | 49894349 | G | A | 0.23 | 0.040 | 0.007 | 1.53E-08 |
| rs6465151 | 7 | 88310899 | T | C | 0.11 | 0.056 | 0.009 | 1.90E-09 |
| rs75932578 | 7 | 106844694 | C | T | 0.78 | 0.040 | 0.007 | 4.15E-08 |
| rs670501 | 7 | 108625185 | T | C | 0.21 | 0.053 | 0.007 | 7.40E-13 |
| rs8180817 | 7 | 114047542 | G | C | 0.57 | 0.049 | 0.006 | 1.83E-16 |
| rs12666306 | 7 | 115082406 | A | G | 0.50 | 0.042 | 0.006 | 2.24E-12 |
| rs17520265 | 7 | 119674508 | G | A | 0.97 | 0.091 | 0.016 | 2.87E-08 |
| rs6967168 | 7 | 132672192 | G | T | 0.25 | 0.044 | 0.007 | 1.39E-10 |
| rs2598293 | 7 | 133989882 | T | C | 0.48 | 0.035 | 0.006 | 2.48E-09 |
| rs1731951 | 7 | 137075847 | T | A | 0.56 | 0.035 | 0.006 | 1.36E-08 |
| rs28611339 | 8 | 10170037 | T | G | 0.13 | 0.058 | 0.009 | 8.46E-11 |
| rs874168 | 8 | 30849450 | T | C | 0.53 | 0.034 | 0.006 | 7.95E-09 |
| rs871994 | 8 | 35190619 | A | C | 0.44 | 0.035 | 0.006 | 5.50E-09 |
| rs671985 | 8 | 60914783 | G | A | 0.55 | 0.038 | 0.006 | 2.79E-10 |
| rs4588900 | 8 | 73890425 | A | G | 0.52 | 0.033 | 0.006 | 1.57E-08 |
| rs17643634 | 8 | 91650818 | C | T | 0.84 | 0.060 | 0.008 | 1.34E-13 |
| rs28552587 | 8 | 103356226 | A | G | 0.56 | 0.033 | 0.006 | 3.30E-08 |
| rs10955647 | 8 | 114154187 | T | G | 0.53 | 0.033 | 0.006 | 1.84E-08 |
| rs2737240 | 8 | 116657235 | A | G | 0.71 | 0.036 | 0.007 | 3.37E-08 |
| rs10758593 | 9 | 4292083 | G | A | 0.60 | 0.036 | 0.006 | 4.90E-09 |
| rs118166957 | 9 | 8858043 | T | C | 0.16 | 0.068 | 0.008 | 1.95E-16 |
| rs10756571 | 9 | 14534505 | T | C | 0.69 | 0.036 | 0.006 | 1.80E-08 |
| rs4090240 | 9 | 77118987 | C | T | 0.72 | 0.039 | 0.007 | 8.46E-09 |
| rs7044885 | 9 | 81739348 | G | C | 0.56 | 0.041 | 0.006 | 5.67E-12 |
| rs10761240 | 9 | 96361922 | G | A | 0.60 | 0.043 | 0.006 | 2.12E-12 |
| rs1927902 | 9 | 120518991 | T | C | 0.25 | 0.053 | 0.007 | 1.15E-14 |
| rs2792990 | 9 | 125621610 | C | G | 0.86 | 0.054 | 0.008 | 1.15E-10 |
| rs6597649 | 9 | 133786652 | T | C | 0.40 | 0.033 | 0.006 | 3.05E-08 |
| rs7040224 | 9 | 134886837 | A | G | 0.32 | 0.037 | 0.006 | 4.24E-09 |
| rs72773790 | 9 | 139109080 | T | C | 0.67 | 0.037 | 0.006 | 3.71E-09 |
| rs77641763 | 9 | 140265782 | T | C | 0.12 | 0.071 | 0.009 | 6.53E-15 |
| rs12251016 | 10 | 21821918 | T | A | 0.34 | 0.039 | 0.006 | 3.89E-10 |
| rs10825503 | 10 | 57177470 | T | G | 0.49 | 0.033 | 0.006 | 1.43E-08 |
| rs224029 | 10 | 64519299 | C | T | 0.60 | 0.039 | 0.006 | 2.51E-10 |
| rs11001276 | 10 | 76825638 | T | A | 0.26 | 0.038 | 0.007 | 2.52E-08 |
| rs7475916 | 10 | 77771194 | G | C | 0.65 | 0.037 | 0.006 | 6.70E-09 |
| rs214934 | 11 | 17193475 | T | A | 0.69 | 0.038 | 0.006 | 3.16E-09 |
| rs72899452 | 11 | 45415577 | T | C | 0.06 | 0.074 | 0.012 | 1.00E-09 |
| rs11605348 | 11 | 47606483 | G | A | 0.65 | 0.045 | 0.006 | 7.01E-13 |
| rs12790660 | 11 | 57667222 | C | T | 0.32 | 0.040 | 0.006 | 4.49E-10 |
| rs4592425 | 11 | 62697813 | T | G | 0.70 | 0.040 | 0.006 | 4.31E-10 |
| rs524859 | 11 | 66041079 | G | A | 0.64 | 0.044 | 0.006 | 1.48E-12 |
| rs566673 | 11 | 66401373 | G | T | 0.46 | 0.039 | 0.006 | 1.18E-10 |
| rs56133505 | 11 | 72348039 | A | G | 0.54 | 0.041 | 0.006 | 5.59E-12 |
| rs10898940 | 11 | 73455292 | A | C | 0.52 | 0.034 | 0.006 | 8.09E-09 |
| rs667730 | 11 | 83277325 | T | C | 0.58 | 0.033 | 0.006 | 2.26E-08 |
| rs2221119 | 11 | 88598444 | C | G | 0.44 | 0.036 | 0.006 | 2.00E-09 |
| rs6589988 | 11 | 99126016 | G | A | 0.32 | 0.038 | 0.006 | 4.70E-09 |
| rs1064939 | 11 | 118396331 | A | T | 0.98 | 0.130 | 0.020 | 2.16E-10 |
| rs647905 | 11 | 121534938 | T | C | 0.54 | 0.033 | 0.006 | 2.87E-08 |
| rs2286729 | 12 | 6873818 | A | G | 0.09 | 0.070 | 0.011 | 5.37E-11 |
| rs1167132 | 12 | 43484487 | T | C | 0.39 | 0.035 | 0.006 | 8.73E-09 |
| rs324017 | 12 | 57487814 | A | C | 0.29 | 0.039 | 0.007 | 1.61E-09 |
| rs61921611 | 12 | 66367726 | C | T | 0.31 | 0.044 | 0.006 | 7.84E-12 |
| rs7486418 | 12 | 84336911 | T | G | 0.66 | 0.041 | 0.006 | 6.84E-11 |
| rs6606731 | 12 | 109982578 | A | T | 0.19 | 0.043 | 0.008 | 1.51E-08 |
| rs4767645 | 12 | 118385788 | G | T | 0.54 | 0.037 | 0.006 | 6.47E-10 |
| rs28582096 | 12 | 123856998 | G | A | 0.80 | 0.054 | 0.007 | 1.74E-13 |
| rs9527083 | 13 | 53991125 | G | A | 0.33 | 0.076 | 0.006 | 1.61E-32 |
| rs1031654 | 13 | 54382035 | C | A | 0.20 | 0.051 | 0.007 | 3.88E-12 |
| rs7992992 | 13 | 54721699 | A | G | 0.13 | 0.051 | 0.009 | 1.15E-08 |
| rs6562066 | 13 | 60532796 | T | C | 0.37 | 0.039 | 0.006 | 1.38E-10 |
| rs9563886 | 13 | 61720066 | C | T | 0.39 | 0.034 | 0.006 | 3.08E-08 |
| rs9540729 | 13 | 66947124 | A | T | 0.48 | 0.036 | 0.006 | 1.40E-09 |
| rs11149313 | 13 | 85294881 | A | G | 0.73 | 0.040 | 0.007 | 2.38E-09 |
| rs2389631 | 13 | 96932868 | C | A | 0.33 | 0.040 | 0.006 | 2.03E-10 |
| rs1536053 | 13 | 111982291 | C | T | 0.68 | 0.038 | 0.006 | 6.04E-09 |
| rs4981170 | 14 | 33412996 | G | A | 0.81 | 0.054 | 0.008 | 7.33E-13 |
| rs12912299 | 15 | 38897857 | C | T | 0.51 | 0.043 | 0.006 | 4.42E-13 |
| rs715338 | 15 | 57215867 | A | G | 0.58 | 0.041 | 0.006 | 7.85E-12 |
| rs7168238 | 15 | 66709386 | C | G | 0.07 | 0.064 | 0.011 | 1.80E-08 |
| rs1038093 | 15 | 74012409 | T | C | 0.63 | 0.039 | 0.006 | 2.47E-10 |
| rs12917449 | 15 | 74331659 | C | A | 0.19 | 0.042 | 0.008 | 2.97E-08 |
| rs176644 | 15 | 89913632 | T | G | 0.40 | 0.035 | 0.006 | 9.49E-09 |
| rs4702 | 15 | 91426560 | G | A | 0.44 | 0.048 | 0.006 | 6.78E-16 |
| rs7402939 | 15 | 99183876 | C | T | 0.62 | 0.036 | 0.006 | 5.19E-09 |
| rs3184470 | 16 | 715164 | G | A | 0.65 | 0.038 | 0.006 | 9.73E-10 |
| rs12924275 | 16 | 9191790 | T | C | 0.27 | 0.038 | 0.007 | 1.93E-08 |
| rs830716 | 16 | 12323509 | C | G | 0.71 | 0.045 | 0.007 | 8.68E-12 |
| rs66674044 | 16 | 19904344 | T | A | 0.14 | 0.060 | 0.009 | 2.18E-12 |
| rs4788203 | 16 | 29978827 | G | A | 0.57 | 0.035 | 0.006 | 6.32E-09 |
| rs1015438 | 16 | 51177517 | A | G | 0.19 | 0.058 | 0.008 | 2.51E-14 |
| rs34214423 | 16 | 52303107 | A | C | 0.81 | 0.045 | 0.008 | 3.18E-09 |
| rs4238755 | 16 | 52746089 | C | A | 0.74 | 0.043 | 0.007 | 2.30E-10 |
| rs9931543 | 16 | 56128782 | T | C | 0.74 | 0.048 | 0.007 | 1.11E-12 |
| rs3902952 | 16 | 61647589 | T | C | 0.19 | 0.048 | 0.008 | 2.55E-10 |
| rs35322724 | 16 | 77137324 | A | C | 0.58 | 0.049 | 0.006 | 3.75E-16 |
| rs62068188 | 17 | 2400876 | T | C | 0.83 | 0.049 | 0.008 | 1.18E-09 |
| rs34490907 | 17 | 26933741 | C | G | 0.89 | 0.054 | 0.009 | 1.76E-08 |
| rs7214267 | 17 | 43157709 | G | A | 0.42 | 0.044 | 0.006 | 5.09E-13 |
| rs11650304 | 17 | 46035001 | C | G | 0.93 | 0.067 | 0.012 | 1.23E-08 |
| rs4643373 | 17 | 47123423 | T | C | 0.70 | 0.041 | 0.007 | 1.58E-10 |
| rs9889282 | 17 | 50259142 | C | A | 0.39 | 0.042 | 0.006 | 4.70E-12 |
| rs8076183 | 17 | 61024696 | C | T | 0.55 | 0.038 | 0.006 | 2.75E-10 |
| rs12454003 | 18 | 26315799 | G | C | 0.52 | 0.035 | 0.006 | 4.94E-09 |
| rs12605642 | 18 | 31313965 | T | G | 0.49 | 0.035 | 0.006 | 2.13E-09 |
| rs10502966 | 18 | 50748499 | G | A | 0.42 | 0.039 | 0.006 | 8.54E-11 |
| rs60565673 | 18 | 52906830 | G | T | 0.38 | 0.043 | 0.006 | 1.59E-12 |
| rs9964420 | 18 | 56824041 | A | C | 0.30 | 0.035 | 0.007 | 4.54E-08 |
| rs12983032 | 19 | 5073447 | G | A | 0.66 | 0.043 | 0.006 | 1.07E-11 |
| rs6510033 | 19 | 30710785 | G | A | 0.27 | 0.037 | 0.007 | 4.66E-08 |
| rs429358 | 19 | 45411941 | T | C | 0.85 | 0.046 | 0.008 | 2.13E-08 |
| rs908668 | 19 | 56134038 | T | C | 0.21 | 0.050 | 0.007 | 1.41E-11 |
| rs6119267 | 20 | 31163914 | G | C | 0.31 | 0.060 | 0.006 | 2.32E-20 |
| rs2867690 | 20 | 41972028 | T | C | 0.18 | 0.042 | 0.008 | 3.70E-08 |
| rs910187 | 20 | 45841052 | G | A | 0.63 | 0.035 | 0.006 | 1.63E-08 |
| rs6019663 | 20 | 47774512 | T | C | 0.29 | 0.040 | 0.007 | 6.47E-10 |
| rs742760 | 20 | 50985290 | A | T | 0.82 | 0.043 | 0.008 | 2.48E-08 |
| rs76145129 | 20 | 62670427 | G | T | 0.88 | 0.050 | 0.009 | 2.73E-08 |
| rs2838787 | 21 | 46539725 | G | A | 0.61 | 0.036 | 0.006 | 7.65E-09 |
| rs11090039 | 22 | 41496800 | A | G | 0.29 | 0.039 | 0.007 | 1.82E-09 |

Chr, chromosome; EA, effect allele; EAF, effect allele frequency; NEA, non-effect allele.

**Supplementary Table 3**. Definition and sources of information for 9 cardiovascular diseases outcomes in UK Biobank

| **Outcome** | **Number of cases** | **ICD-9 diagnosis** | **ICD-10 diagnosis** | **OPCS procedure** | **Self-report†** |
| --- | --- | --- | --- | --- | --- |
|  |  |  |  |  |  |
| Coronary artery disease | 29 278 | 410, 411, 412, 414.0, 414.8, 414.9 | I21, I22, I23, I24, I25.1, I25.2, I25.5, I25.6, I25.8, I25.9 | K40, K41, K42, K43, K44, K45, K46, K49, K50.1, K50.2, K50.4, K75 | 20002, 20004, 6150 |
| Heart failure | 6712 | 402.01, 402.11, 402.91, 404.01, 404.11, 404.91, 404.03, 404.13, 404.93, 428 | I11.0, I13.0, I13.2, I50 |  | 20002 |
| Atrial fibrillation | 16 945 | 427.3 | I48 |  | 20002 |
| Aortic valve stenosis | 2244 |  | I35.0, I35.2 |  | 20002 |
| Abdominal aortic aneurysm | 1094 | 441.3, 441.4 | I71.3, I71.4 | L19.4, L19.5 | 20002 |
| Ischemic stroke | 4602 | 434, 436 | I63, I64 |  | 20002 |
| Haemorrhagic stroke | 2148 | 430, 431 | I60, I61 |  | 20002 |
| Peripheral arterial disease | 3415 | 443.8, 443.9 | I73.8, I73.9 |  | 20002 |
| Venous thromboembolism | 15602 | 415.1, 451.1, 452, 453.0, 453.4, 453.9 | I26, I80.1, I80.2, I81, I82.0 | L90.2 | 20002, 6152 |

ICD, International Classification of Disease; OPCS, Office of Population Censuses and Surveys Classification of Surgical Operations and Procedures.

*Follow-up for incident cases was until March 31, 2017 and date of death was recorded until February 14, 2018.

†Numbers refer to data codes used in UK Biobank: 6150/6152 = Health condition diagnosed by doctor (self-reported); 6177 = Medication for health condition (self-reported); 20002 = Non-cancer illness code (self-reported from interview with nurse); 20004 = Surgical operation code.

**Supplementary Table 4**. Associations of genetically predicted liability to insomnia with 9 individual cardiovascular diseases in UK Biobank in sensitivity analyses

| **Cardiovascular disease** | **Weighted median method** | | |  | **MR-Egger regression method** | | | | |
| --- | --- | --- | --- | --- | --- | --- | --- | --- | --- |
|  | **OR** | **95% CI** | ***p*** |  | **OR** | **95% CI** | ***p*** | **Intercept** | ***p*** |
| Abdominal aortic aneurysm | 1.24 | 0.98,1.56 | 0.073 |  | 1.15 | 0.63,2.11 | 0.646 | -0.001 | 0.965 |
| Atrial fibrillation | 1.08 | 1.02,1.15 | 0.013 |  | 0.98 | 0.82,1.18 | 0.868 | 0.005 | 0.218 |
| Aortic valve stenosis | 1.02 | 0.87,1.20 | 0.810 |  | 1.01 | 0.63,1.61 | 0.982 | 0.007 | 0.508 |
| Coronary artery disease | 1.18 | 1.12,1.24 | 5.36×10^-11^ |  | 1.08 | 0.90,1.29 | 0.433 | 0.005 | 0.264 |
| Heart failure | 1.18 | 1.07,1.29 | 0.001 |  | 1.23 | 0.94,1.61 | 0.139 | -0.001 | 0.927 |
| Peripheral arterial disease | 1.22 | 1.07,1.40 | 0.004 |  | 1.24 | 0.88,1.75 | 0.218 | -0.001 | 0.920 |
| Haemorrhagic stroke | 1.09 | 0.92,1.29 | 0.313 |  | 2.06 | 1.25,3.39 | 0.005 | -0.027 | 0.016 |
| Ischemic stroke | 1.20 | 1.08,1.34 | 0.001 |  | 0.85 | 0.62,1.18 | 0.342 | 0.013 | 0.066 |
| Venous thromboembolism | 1.13 | 1.05,1.20 | 3.53×10^-4^ |  | 0.87 | 0.71,1.08 | 0.204 | 0.011 | 0.012 |

CI indicates confidence interval; OR, odds ratio.

There was possible pleiotropy in the associations for haemorrhagic stroke and venous thromboembolism.

**Supplementary Figure 1.** Associations of genetically predicted liability to depression with 9 individual cardiovascular diseases in UK Biobank

**
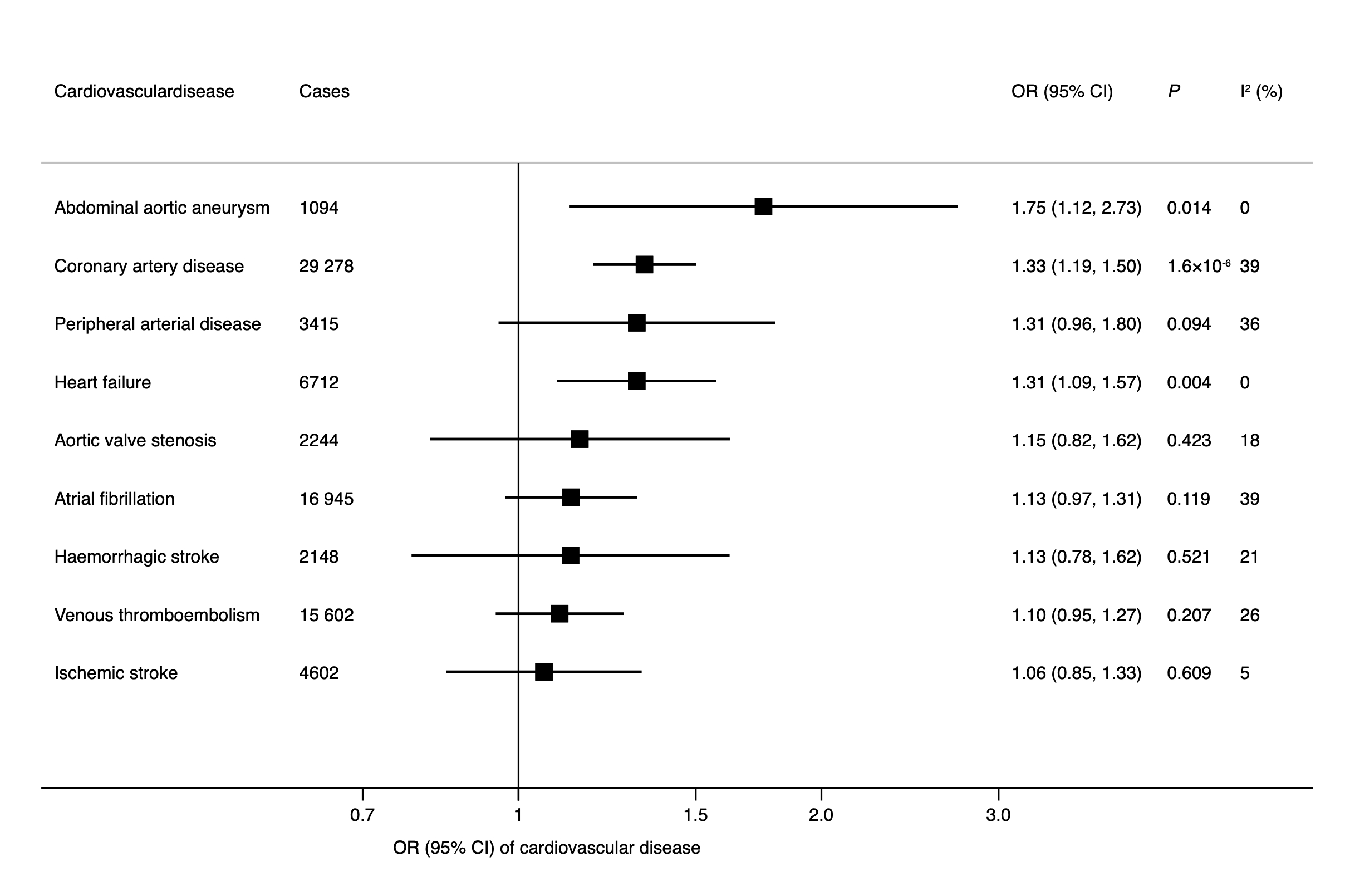
**

CI indicates confidence interval; OR, odds ratio.

Odds ratios are expressed per genetically predicted 1-unit-higher log-odds of liability to depression. Estimates are from the multiplicative random-effects inverse variance-weighted method. The *I*^2^ statistic quantifies the amount of heterogeneity among estimates based on individual SNPs. Significant at the Bonferroni-corrected threshold of *P*< 0.006.

**Supplementary Figure 2.** Associations of genetically predicted liability to insomnia with 9 individual cardiovascular diseases after adjusting for depression in UK Biobank

**
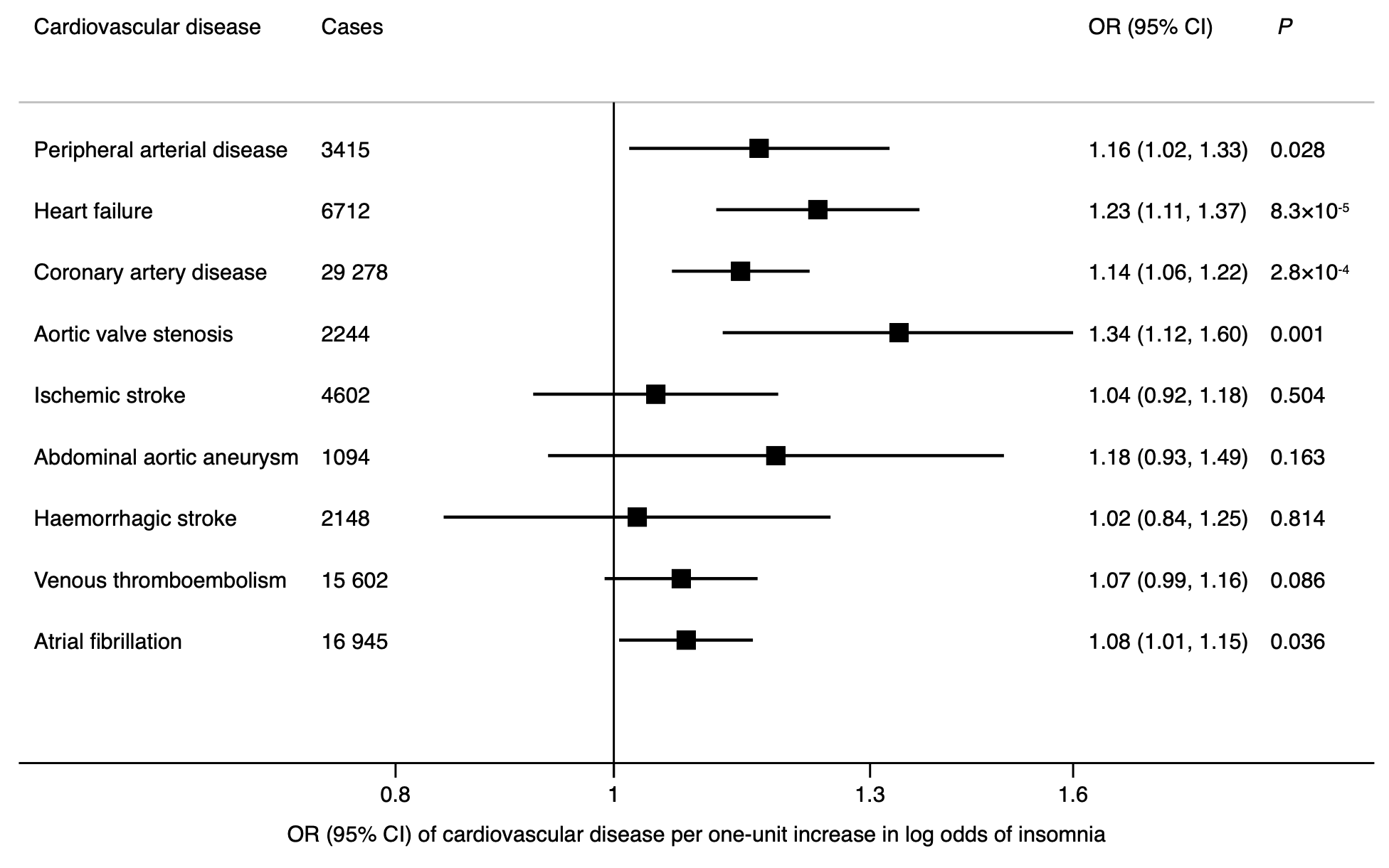
**

CI indicates confidence interval; OR, odds ratio.

Odds ratios are expressed per genetically predicted 1-unit-higher log-odds of liability to insomnia. Estimates are from the multivariable multiplicative random-effects inverse variance-weighted method.

**Supplementary Figure 3.** Associations of genetically predicted liability to insomnia with 9 individual cardiovascular diseases after adjusting for sleep duration in UK Biobank


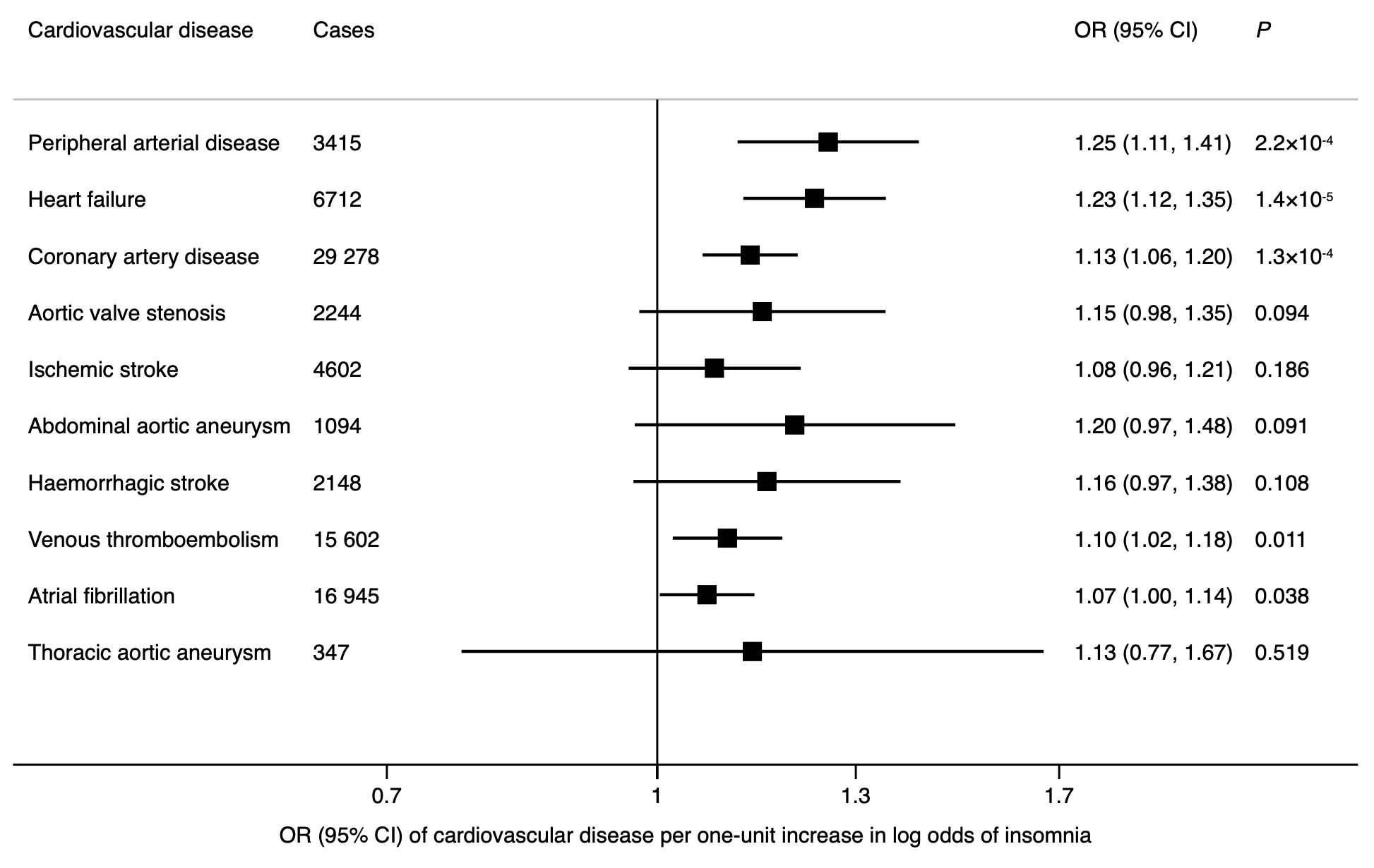


CI, confidence interval; OR, odds ratio.

Odds ratios are expressed per genetically predicted 1-unit-higher log-odds of liability to insomnia. Estimates are from the multivariable multiplicative random-effects inverse variance-weighted method.

**Supplementary Figure 4.** Associations of genetically predicted liability to insomnia with body mass index, smoking, alcohol drinking and type 2 diabetes mellitus


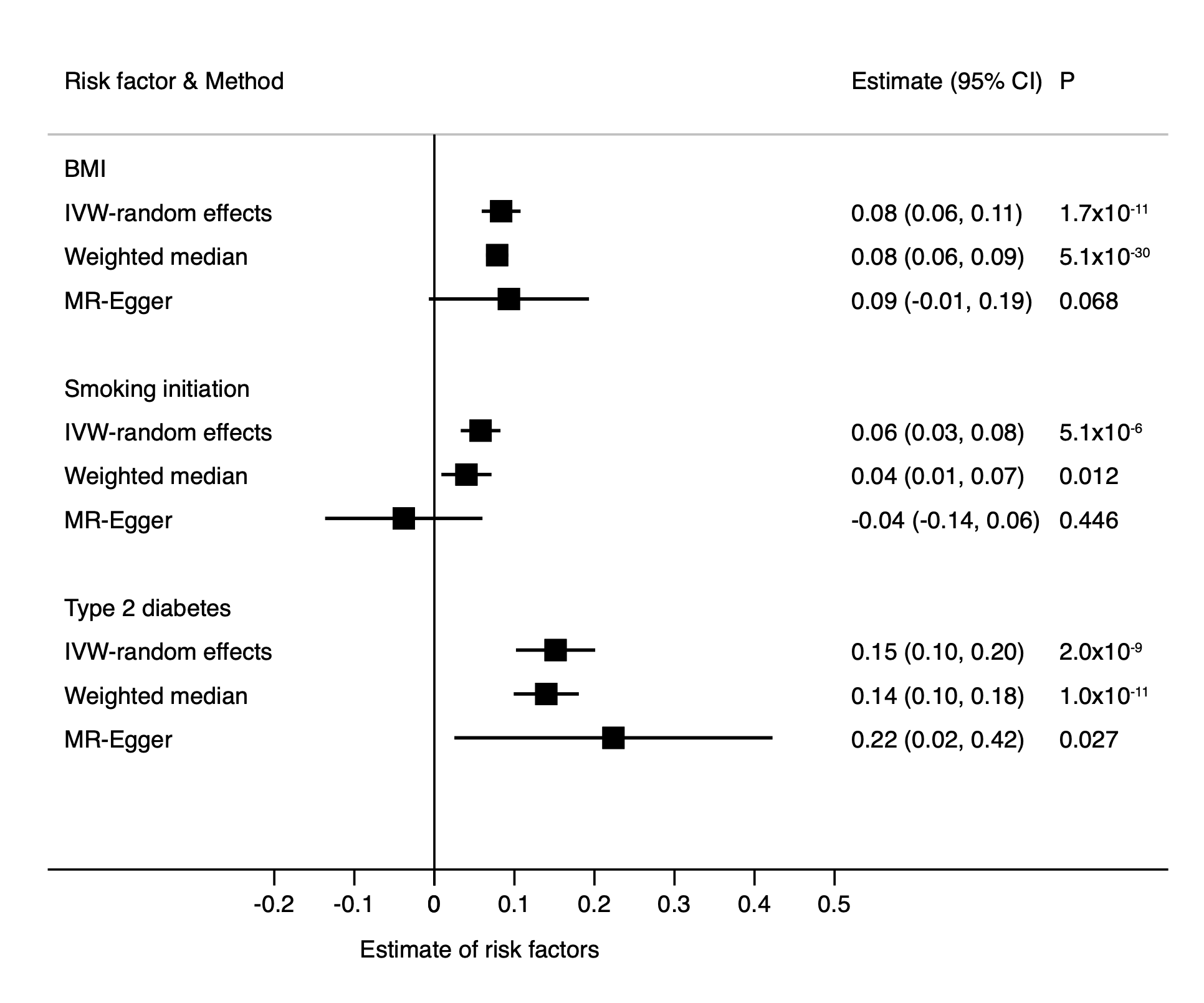


CI indicates confidence interval; OR, odds ratio.

Odds ratios are expressed per genetically predicted 1-unit-higher log-odds of liability to insomnia. Estimates are from the multiplicative random-effects inverse variance-weighted method.

**Supplementary Figure 5.** Associations of genetically predicted liability to insomnia with 9 individual cardiovascular diseases after adjusting for body mass index, smoking and type 2 diabetes mellitus in UK Biobank


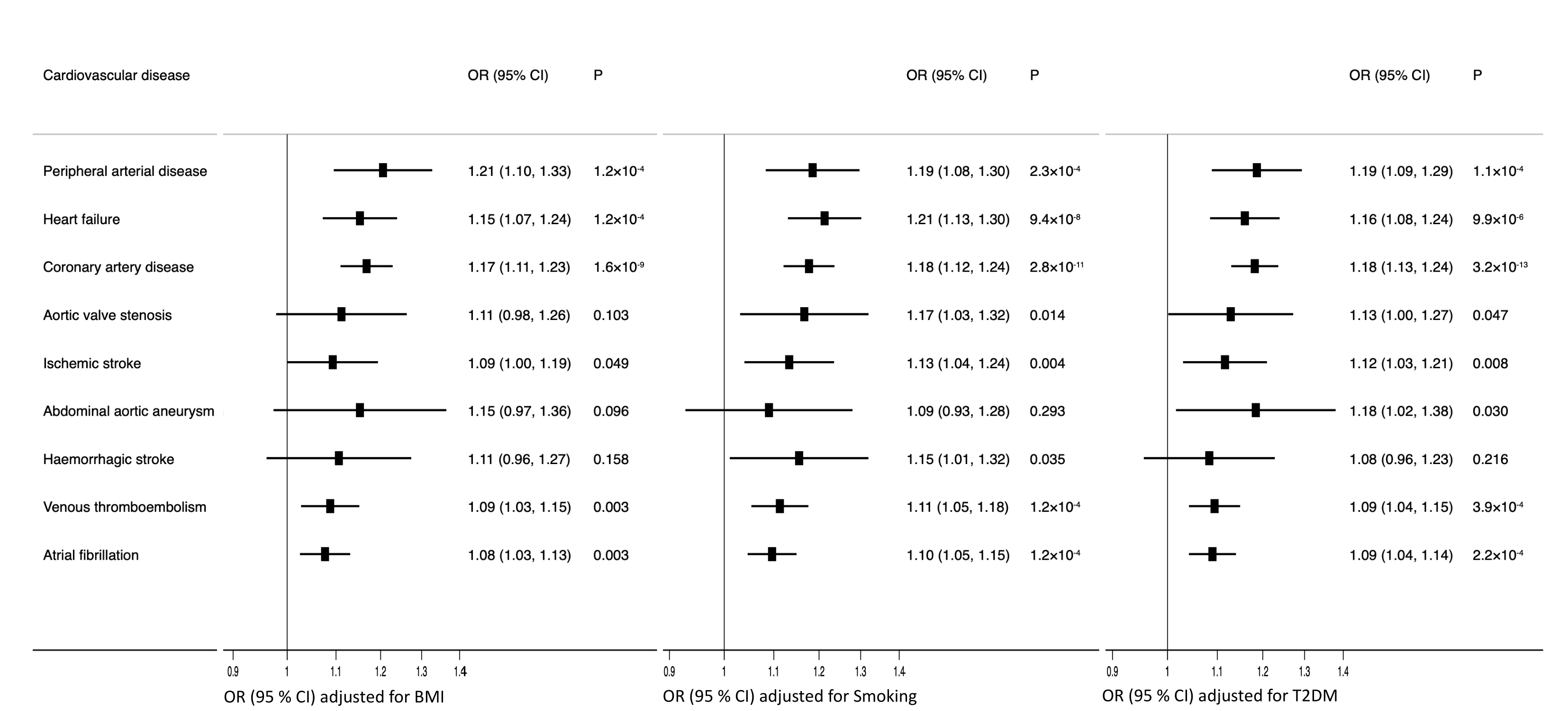


BMI indicates body mass index; CI, confidence interval; OR, odds ratio; T2DM, type 2 diabetes.

Odds ratios are expressed per genetically predicted 1-unit-higher log-odds of liability to insomnia. Estimates are from the multivariable multiplicative random-effects inverse variance-weighted method.
